# Orbital Venous Varices: A Rare Bilateral Asymptomatic Presentation

**DOI:** 10.7759/cureus.3302

**Published:** 2018-09-14

**Authors:** Alexandros Pappas, Julio M Araque, Vimal Sarup

**Affiliations:** 1 Department of Ophthalmology, Howard University Hospital, Washington, D.C, USA; 2 Department of Radiology, Orlando Veterans Affairs Medical Center, Orlando, USA; 3 Department of Ophthalmology, University of Central Florida College of Medicine, Galveston, USA

**Keywords:** orbital masses, orbital varices, orbital varix, bilateral orbital varices, varix, varices, orbit, orbital venous varices, orbital veins, orbital venous varix

## Abstract

This report illustrates a case of asymptomatic bilateral orbital varices in a 64-year-old Caucasian male. The orbital varices were incidentally discovered while investigating the patient’s initial presentation of bilateral chorioretinal scars and optic nerve head drusen. Magnetic resonance imaging (MRI) of the brain and orbits with contrast confirmed the presence of bilateral varices of the inferior ophthalmic veins and pterygoid plexuses. The occurrence of bilateral orbital varices is quite rare, and few asymptomatic cases have been described in the literature.

## Introduction

Orbital varices typically result from a congenital weakness in the postcapillary venous wall [1]. This can lead to the proliferation and dramatic dilation of the valveless orbital veins. These varices distend during maneuvers that increase venous pressure depending on the extent of communication with the venous system. Patients typically present with unilateral stress proptosis that manifests with activities that increase venous pressure (coughing, crying, bending, straining, breath holding, or Valsalva maneuver) [2]. We report a case of an asymptomatic patient who had bilateral varices of the inferior ophthalmic veins and pterygoid venous plexuses.

## Case presentation

A 64-year-old Caucasian male with a medical history of type 2 diabetes mellitus, hypertension, and hyperlipidemia presented to an eye clinic for a diabetic eye exam. He had no ocular complaints aside from slightly blurred vision, which he attributed to “scarring on his retina.” He stated that he had developed “smoky vision” several years ago, which had been treated with oral and topical medications. He denied any current ocular discomfort or pain.

On exam, his visual acuity was 20/25^+2^ in the right eye and 20/20^-2^ in the left with correction. Anterior segment examination did not reveal any abnormalities. Intraocular pressures were within normal limits. Dilated fundus examination found optic nerve head drusen, extramacular healed chorioretinal scars, and mild non-proliferative diabetic retinopathy in both eyes.

**Figure 1:**
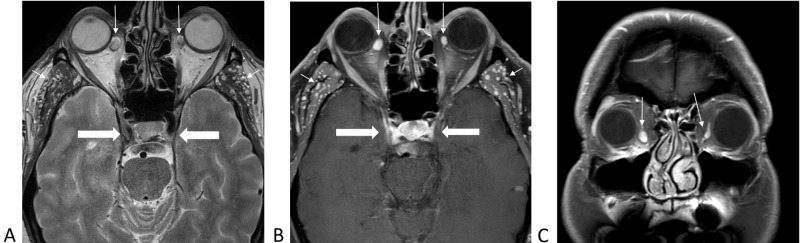
Magnetic resonance imaging of the brain demonstrating varices of inferior ophthalmic veins bilaterally (long arrows), infratemporal venous vessels (short arrows), and normal cavernous sinuses (thick arrows). 1A, axial T2-weighted without contrast. 1B, axial T1-weighted with contrast and fat saturation. 1C, coronal T1-weighted with contrast and fat saturation.

**Figure 2:**
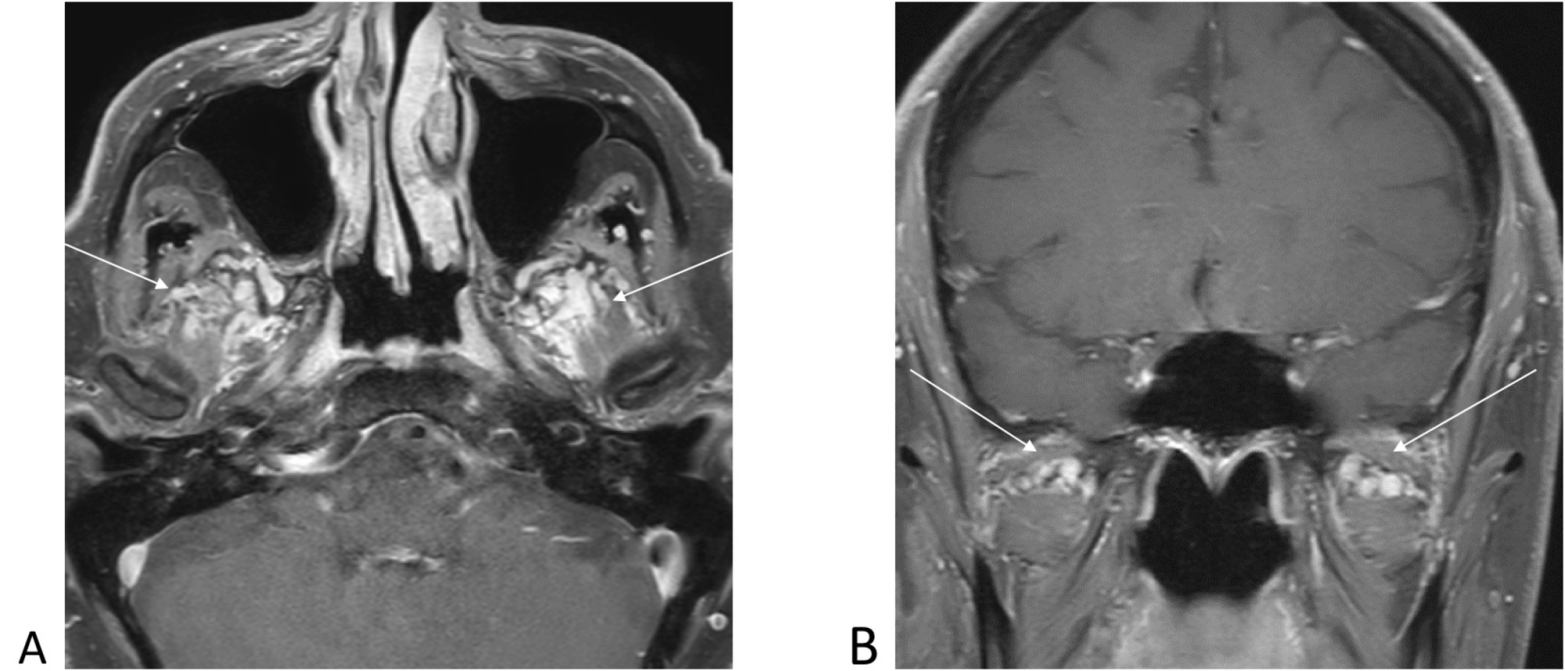
Post-contrast T1-weighted magnetic resonance images demonstrating prominent pterygoid plexus varices noted bilaterally (arrows). 2A, axial T1-weighted with contrast. 2B, coronal T1-weighted with contrast.

**Figure 3:**
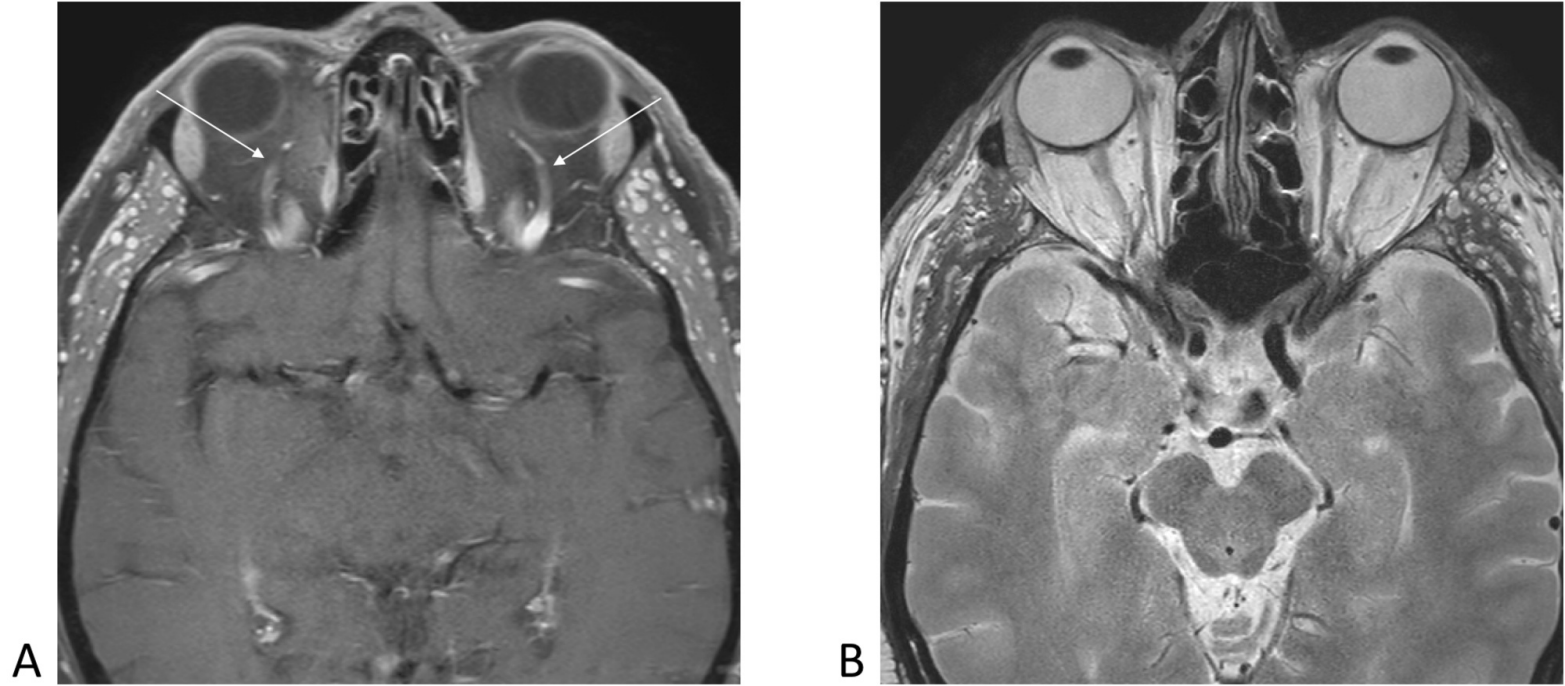
Axial magnetic resonance images demonstrating normal appearance of bilateral superior ophthalmic veins (arrows) and normal morphology of eyeballs and optic nerves bilaterally. 3A, axial T1-weighted with contrast. 3B, axial T2-weighted without contrast.

Diagnostic work-up included laboratory testing and magnetic resonance imaging (MRI) of the brain and orbits with and without contrast. This work-up was prompted by the history of chorioretinal scarring and blurry vision in the past requiring treatment. Laboratory workup was negative aside from positive toxoplasma IgG antibodies. MRI of the brain and orbits with and without contrast revealed varices of the bilateral inferior ophthalmic veins, bilateral pterygoid plexuses, and the infratemporal veins. There was no dilation or thrombosis of the superior ophthalmic veins, and no abnormal enhancing mass lesions within the orbits or brain parenchyma. No intracranial arteriovenous malformations, dural fistulas, or carotid cavernous fistulas were identified (Figures 1-3).

## Discussion

A venous varix is a dilation of one or more veins associated with low blood flow and distensible walls. Orbital varices are a rare condition, accounting for less than 1.3% of all orbital masses [3-4]. Although most cases are typically unilateral, our case represents a rare presentation of bilateral orbital varices.

The orbital venous system is highly variable compared to other venous systems elsewhere in the body. The inferior ophthalmic venous plexus has multiple valveless interconnected veins with extremely small diameters and short lengths [5]. The orbital venous system receives contributing veins from multiple tributaries, including the palpebral, vortex, lacrimal, muscular, ethmoidal, and, in some instances, the central retinal vein [5-6]. The pterygoid plexus specifically is connected to the inferior orbital venous system via communicating vessels that travel through the inferior orbital fissure [5].

Orbital varices can be divided into primary and secondary orbital varices. Primary orbital varices are idiopathic and confined to the orbit. Secondary orbital varices are acquired, and usually a result of a carotid-cavernous fistula, dural arteriovenous fistulas, or intracranial arteriovenous malformations [3-4].

Primary orbital vascular malformations are believed to be present at birth. Even though it is thought to be congenital, patients do not typically become symptomatic until they are young adults. However, cases have been reported across all age groups [3-4]. These lesions usually present with intermittent diplopia and/or proptosis during episodes of straining (Valsalva) or specific positionings such as prone positioning or stooping. Over time, the long-lasting distension can create more room for the globe to “fall back” when the varix is deflated, leading to paradoxical enophthalmos at rest [1,4].

In addition to the typical presentations, many patients will be diagnosed due to a sudden complication. Orbital venous varices are the most common cause of spontaneous intraorbital hemorrhage [1]. Acute thrombosis is another potential complication, where patients present with acute onset of retro-orbital pain, proptosis, and decreased visual acuity [1,3]. In rare circumstances, larger lesions involving the superior ophthalmic vein may present as a lacrimal mass [4,7]. The differential diagnosis includes other orbital venous malformations such as varicocele, venous angioma, or lymphangioma.

Imaging modalities to detect varices include ultrasound, color Doppler imaging, computerized tomography (CT), and MRI including magnetic resonance venography (MRV). During imaging, dynamic maneuvers such as Valsalva, in conjunction with specific positioning, can aid in the visualization of varix [2]. Orbital CT may easily delineate varices and calcified phleboliths, which occur due to thrombus formation. In some instances, orbital varices can erode the surrounding bony structures, leading to orbital expansion and osseous defects of the orbit [8].

For asymptomatic varices, observation with serial imaging is the usual method of follow-up. This reported patient will be monitored and imaged periodically. For symptomatic varices causing a mass effect, disfigurement, vision loss, optic nerve compression, thrombosis, or hemorrhage, surgical intervention is warranted with a multidisciplinary approach. Generally, orbitotomy with excision of the varices is the treatment of choice. Varices are easier to excise with less invasive modalities if the anterior portion is thrombosed, with identification being more difficult in a supine patient with non-thrombosed varix [3-4]. Recurrence in these cases is usually due to subtotal excision. To avoid this, the surgeon should always aim to resect or clip varix as far back toward the orbital apex as possible [4,7]. Varices can also be embolized for easier identification and to reduce bleeding during surgery. Embolization techniques include the use of micro-coils, as well as the injection of glue, onyx, or cyanoacrylate [3-4,7,9]. These techniques are more beneficial in posterior or deeper lesions.

## Conclusions

There can be significant variations in the vascular pattern of orbits between individuals. This case report illustrates the extremely rare combination of bilateral orbital varices in a completely asymptomatic individual.
